# Proximal Tibial Epiphysis Fracture in a 13-Year-Old Male Athlete

**DOI:** 10.1155/2017/4823589

**Published:** 2017-05-21

**Authors:** Ioannis M. Stavrakakis, Pavlos E. Katsoulis, Maria S. Katsafarou

**Affiliations:** Department of Orthopaedics and Traumatology, General Hospital of Agios Nikolaos, Knosou 4, 721 00 Crete, Greece

## Abstract

Fractures of the proximal epiphysis of the tibia are rare, representing 0.5 to 3.0% of all epiphyseal injuries. These injuries can damage the popliteal vessels and their bifurcation, affecting the blood supply of the lower limb, as well as the nerves below the knee. Epiphyseal growth arrest is also a potential complication, leading to various angular deformities. We present a case of a 13-year-old male athlete with a posteriorly displaced Salter-Harris type II fracture of the proximal epiphysis of the left tibia who was treated conservatively with closed reduction and cast immobilization.

## 1. Introduction

Tibial tuberosity avulsion fractures are rare. This rarity is due to the anatomy of the collateral ligaments which are inserted distally into the metaphysis protecting the epiphysis. This injury can threaten the limb circulation, secondary to vascular compromise or compartment syndrome, and it should be treated as an urgent situation. The goal of treatment is anatomical reduction and stabilisation in order to prevent significant soft tissue injury, malunion, and growth arrest [1].

## 2. Case Presentation

A 13-year-old male athlete presented to the emergency department with an injured left tibia. The patient reported a left knee forced hyperflexion during a football match, which caused the injury. The described mechanism of injury was violent contraction of the quadriceps muscle against a fixed tibia.

The left knee was held in 100° of flexion with complete inability to extend the knee or bear weight. On examination, there was obvious swelling over the proximal tibia and on palpation there was tenderness on the anterolateral part of the proximal end of the tibia. No neurovascular impairment was identified. The patient was able to actively move his ankle, as well as his toes, and both posterior tibialis artery and dorsalis pedis artery were palpable.

**Figure 1:**
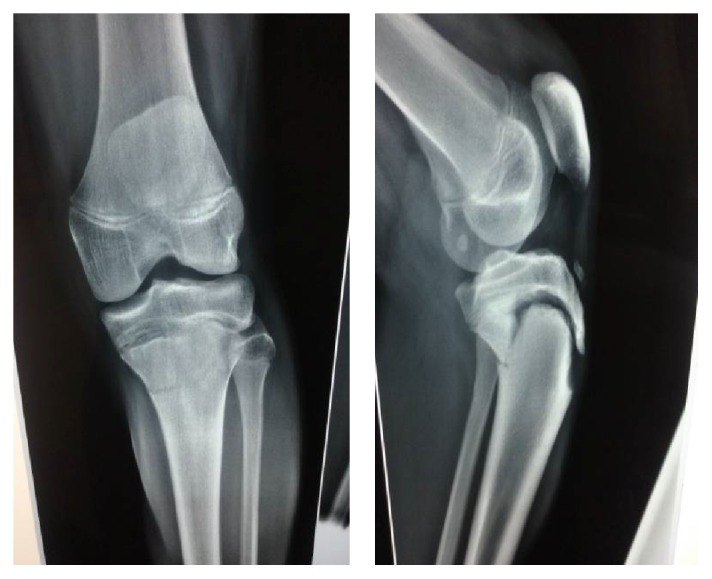
Initial injury.

From the plain radiographs, a displaced flexion type Salter-Harris type II fracture of the proximal tibia epiphysis was identified (Figure 1).

**Figure 2:**
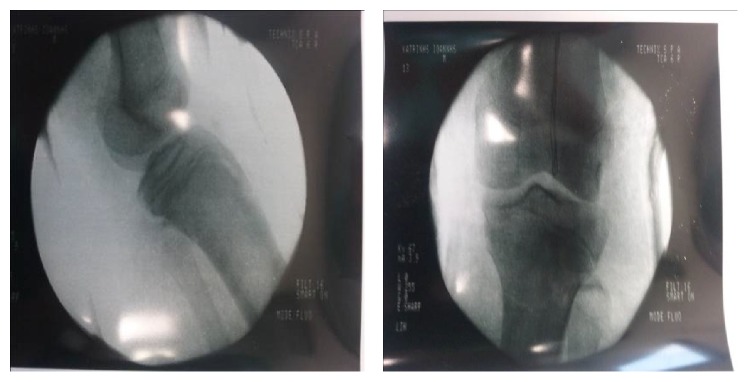
Fluoroscopic image showing successful closed reduction of the fracture.

Few hours after admission, the patient was led to the theatre and, under general anesthesia, a closed reduction of the fracture was performed by extending the knee and putting strong pressure over the tibial tubercle. Reduction was confirmed using image intensifier (Figure 2) and stability of the fracture was verified, as there was no displacement at 80° of knee flexion. After reduction, the dorsalis pedis artery and the posterior tibial artery were palpable. Since there was no displacement with that degree of flexion, percutaneous pinning of the fracture was not performed and only a circumferential cast with the knee in full extension was applied.

**Figure 3:**
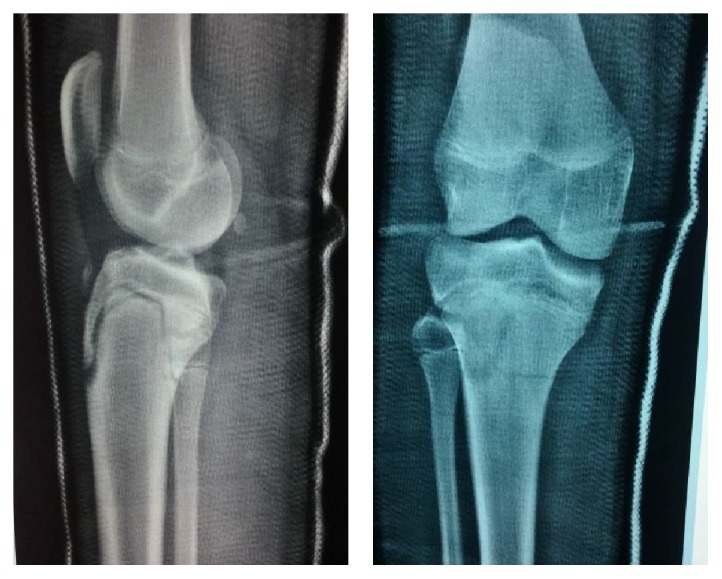
Plain radiographs one week after reduction.

**Figure 4:**
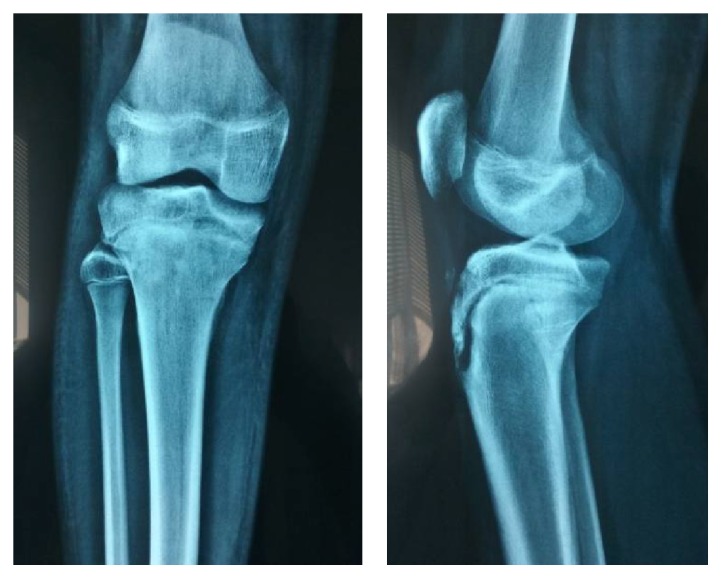
Plain radiographs 6 weeks after reduction.

Patient was hospitalized in the Orthopaedic Department under close observation of limb neurovascular status. On the same night after reduction, the patient developed severe pain, which subsided after splitting the cast. The next few days, the patient developed severe swelling over the anterior tibia compartment, but there were no clinical signs of compartment syndrome. Posterior tibialis remained easily palpable, whereas the dorsalis pedis became weak but easily identifiable with the Doppler ultrasound scan. Fracture remained in a satisfactory position one week after reduction (Figure 3) and a full femur-tibia-ankle cast in 5° of knee flexion was applied. 2 weeks after injury, radiographs of the fracture were still satisfactory. 6 weeks after injury, fracture position was satisfactory with radiographic evidence of healing (Figure 4) and the cast was removed, encouraging active range of motion of the knee [2]. At the same time, the anterior tibialis artery was easily palpable and a triplex ultrasound scan of the lower limb was normal. 8 weeks after injury, the patient achieved full range of motion, and full weight bearing was allowed. MRI scan was performed at this point; as occasionally, these fractures are associated with ligamentous injuries around the knee. MRI scan was normal regarding the cruciate and the collateral ligaments as well as the menisci. Patient's follow-up will continue with serial radiographs at 4 months, 8 months, and 12 months in order to detect early signs of growth arrest or angular deformity and genu recurvatum.

## 3. Discussion

Avulsion fractures of the tibial tubercle and their expansion to the tibial epiphysis are rare. These injuries can damage the vascular supply of the limb, making close monitoring of limb perfusion crucial [3]. An avulsion force, while the quadriceps femoris is contracted, usually separates the anterior portion of the proximal tibial epiphysis (tibial tubercle) [1, 4]. Closure of the proximal tibial physis starts posteriorly and the anterior part fuses lastly. This characteristic explains why this type of fracture affects mainly adolescents and young people between 14 and 18 years of age whose anterior portion of the proximal tibial epiphysis is more vulnerable and is predisposed to type 1 or type 2 Salter-Harris injuries [1].

**Figure 5:**
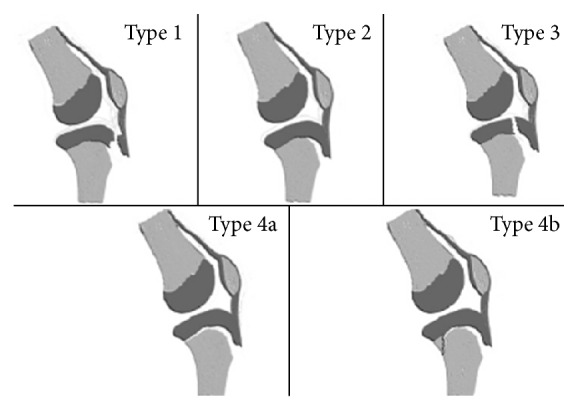
Classification of tibial tuberosity avulsion fractures.

Ogden et al. [5] described three types of tibial tuberosity avulsion fractures: type I, where small fragment is displaced upwards, type II, where entire tongue formed by the tibial tuberosity is hinged upwards, and type III, where the line of fracture passes upwards and backwards across the proximal articular surface of the tibia. Ryu and Debenham [6] expanded the classification including a type IV injury, which involves an avulsion fracture of the proximal tibial tubercle which spreads backwards along the epiphyseal plate (Figure 5).

Our case belongs to the type IV injury with some interesting characteristics regarding limb vascular status. It is well known that a hyperextension injury that results in posterior displacement of the tibia shaft may cause vascular compromise [3, 7]. However, an avulsion injury of the proximal tibial epiphysis, similar to the presented case, may have less potential to damage the limb's vascular supply, because the periosteum on the posterior surface of the tibia remains intact and protects the popliteal vessels [8].

Another cause of poor outcome of the fractures of the proximal tibial epiphysis is physis growth arrest, which can lead to limb length discrepancy or angular deformity, particularly genu recurvatum in flexion type tibial tuberosity avulsion fractures [9]. Gentle anatomical reduction and stabilisation of these fractures with the least physis violation are the main goal of the treatment.

There is controversy in the literature regarding treatment of type IV avulsion tibia tuberosity fractures. Some authors suggest that closed manipulation and percutaneous pinning should be the best option [1, 2, 9, 10], whereas others propose closed manipulation and cast immobilization in extension, provided that the reduction is stable [8, 11]. Inoue et al. described 4 cases of flexion type tibial epiphysis fracture, which were treated with closed reduction and cast immobilization and no major complications occurred [8]. Vyas et al. presented 5 cases with the same type of fracture that were treated uneventfully by conservative means and they concluded that these injuries can be safely managed with closed reduction and cast immobilization in a position of knee extension [11]. Other authors (Blanks et al. and Sułko et al.) also presented good results after conservative treatment of flexion type proximal tibial epiphysis fractures and they proposed closed reduction and cast immobilization as an essential management for this type of injury [12, 13].

In our case, no displacement occurred at 80° of knee flexion after closed reduction, and thus percutaneous pinning was not performed, as pins can potentially violate the physis. Fracture remained in a satisfactory position until healing 6 weeks after manipulation. We think that although pins can secure the reduction, it is not absolutely necessary to apply them, unless the fracture is unstable in flexion or neurovascular impairment occurs.

## 4. Conclusion

This is a case of a young adolescent athlete with a flexion type displaced proximal epiphyseal fracture of the left tibia. The patient was treated with a closed reduction and cast immobilization in extension and the initial outcome is so far satisfactory, with no complications such as neurovascular impairment, compartment syndrome, or redisplacement. Fracture healing in a good position and full range of motion were achieved 8 weeks after injury. His follow-up, in terms of growth arrest or angular deformity, will continue until skeletal maturity.
